# GPC1 exosome and its regulatory miRNAs are specific markers for the detection and target therapy of colorectal cancer

**DOI:** 10.1111/jcmm.12941

**Published:** 2017-02-24

**Authors:** Jian Li, Yuxiang Chen, Xiong Guo, Lin Zhou, Zeming Jia, Zha Peng, Yaping Tang, Weidong Liu, Bin Zhu, Lei Wang, Caiping Ren

**Affiliations:** ^1^ Hepatobiliary and Enteric Surgery Research CenterXiangya HospitalCentral South UniversityChangshaHunanChina; ^2^ School of Pharmaceutical ScienceCentral South UniversityChangshaHunanChina; ^3^ Cancer Research InstituteCollaborative Innovation Center for Cancer MedicineKey Laboratory for Carcinogenesis of Chinese Ministry of HealthSchool of Basic Medical ScienceCentral South UniversityChangshaHunanChina

**Keywords:** colorectal cancer, exosome, biomarker, GPC1, miRNA

## Abstract

Colorectal cancer (CRC) is the second leading cause of cancer‐related deaths worldwide. However, a biomarker for a sensitive and simple diagnostic test and highly effective target therapy of CRC is still clinically unavailable. This study is to investigate the evidence and significance of plasma GPC1 positive exosomes as a biomarker of CRC. Results showed that GPC1^+^ exosomes were successfully isolated from tissues and plasma. The percentage of GPC1^+^ exosomes and the GPC1 protein expression in exosomes from tumour tissues and plasma of CRC patients before surgical treatment was significantly elevated compared to that in the peritumoural tissues and the plasma of healthy controls. miR‐96‐5p and miR‐149 expression in tumour tissues and plasma of CRC patients as well as in the GPC1^+^ exosomes from CRC patients were significantly decreased compared to that in the peritumoural tissues and the plasma of healthy controls. Two months after surgical treatment, levels of all tested markers significantly normalized. Overexpression of miR‐96‐5p and miR‐149 significantly decreased GPC1 expression in HT‐29 and HCT‐116 cells, xenograft tumours, plasma in mice bearing HT‐29 and HCT‐116 tumours, and the secretion of GPC1^+^ exosomes from the HT‐29 and HCT‐116 cells and xenograft tumours. Overexpression of miR‐96‐5p and miR‐149 significantly decreased cell viability and increased cell apoptosis in HT‐29 and HCT‐116 cells, and inhibited the growth of xenograft HT‐29 and HCT‐116 tumours. In conclusion, the increased plasma GPC1^+^ exosomes and reduced plasma miR‐96‐5p and miR‐149 expression are specific markers for the diagnosis of CRC and targets for the therapy of CRC.

## Introduction

Colorectal cancer (CRC) is a common gastrointestinal cancer with high morbidity in Western Europe, North America, Australia and New Zealand 1. Although considerable progress has been made in the surgical treatment and chemotherapy of CRC, as well as in the screening for the disease, CRC is still the second leading cause of cancer‐related deaths worldwide 2, 3. Currently, individualized therapy is becoming a major clinical treatment in CRC, and clinicians are becoming increasingly interested in the impacts of genetics and molecular biology on the development of CRC 4. Therefore, it is important to identify new biomarkers for the early detection, targeting therapy, and prognostic indication in CRC.

Glypican‐1 (GPC1) is a member of the heparan sulphate proteoglycan family and a widespread cell surface protein. Previous studies have observed GPC1 overexpression in several malignancies, including pancreatic cancer, breast cancer and glioma as well as its involvement in tumour growth and angiogenesis in these tumours 5, 6, 7. For example, GPC1 is highly expressed in the vascular endothelial cells of human glioma, but its expression in normal endothelial cells of the brain is barely detectable 8. A previous study demonstrated that GPC1 acts as a co‐receptor of fibroblast growth factor (FGF) to enhance the binding of FGF to its receptor (FGFR), and subsequently promotes FGF‐FGFR activation and signalling 9. A recent study found that GPC1 is specifically enriched in tumour cell‐derived exosomes. The detection of GPC1 in pancreatic cancer exosomes exhibited a high specificity and sensitivity, which can be used to distinguish between patients with benign pancreatic disease and healthy individuals and between advanced‐stage pancreatic cancer and early‐stage pancreatic cancer 10. The level of GPC1 positive exosomes is closely related to survival after surgery in patients with pancreatic cancer 10. These findings suggest that GPC1 positive exosomes may be a tumour‐specific marker, and may closely relate to the occurrence, development and prognosis of tumours.

A recent study compared the genome‐wide expression profiling between the azoxymethane/dextran sulphate sodium salt‐induced CRC tumour tissues and normal colon mucosa in animals and found that GPC1 expression was significantly upregulated while GPC3 was significantly downregulated in murine colorectal adenocarcinoma as well as in human CRC samples 11. However, the roles of GPC1 in early diagnosis, targeted therapy, and prognosis of CRC have not been addressed. In addition, the regulation mechanism of GPC1 expression in CRC is unclear. Previous studies in pancreatic cancer have demonstrated that miR‐96‐5p suppressed GPC1 expression, thereby inhibiting the proliferation of pancreatic cancer cells, and high miR‐96‐5p expression is a predictive marker for good prognosis of pancreatic cancer 12. Another study found that miRNA‐149 is located in the intron of GPC1 and regulates GPC1 expression, which subsequently regulates the angiogenesis response of FGF2 in human endothelial cells 13. In contrast, miR‐182 can promote the growth of CRC cells and tumour invasion 14, 15. Therefore, we hypothesize that these miRNAs may regulate the expression of GPC1.

In this study, we investigated the level of GPC1 positive exosomes and the expression of miR‐96‐5p, miR‐149 and miR‐182‐5p in tumour tissues and plasma of CRC patients before and after surgical treatments. We further determined the regulatory effect of miR‐96‐5p and miR‐149 on the expression of GPC1 and the secretion of GPC1 positive exosomes and subsequent biological functions *in vitro* and *in vivo*.

## Materials and methods

### Sample collection

One hundred and two tumour tissues as well as 89 normal colon tissues were collected from 102 patients with stage I and II CRC who underwent surgical resection of primary tumour and regional lymph nodes. Among the 102 CRC patients, 60 were male and 42 were female with a mean age of 52.4 years. No chemotherapy or radiotherapy was initiated before and until 2 months after surgery. Ten ml of peripheral fasting blood samples were collected from the 102 CRC patients before and 2 months after surgical treatment and from 80 age and gender matched healthy individuals at the Xioangya Hospital, Central South University from September, 2014 to October, 2015. Tissue and plasma samples were immediately frozen at −80°C. This study was pre‐approved by the Ethics Committee of Human Study of Xiangya Hospital and informed written consent was obtained from all patients and control subjects.

### Isolation of plasma from blood

Ten ml of peripheral fasting blood samples were collected into an ethylenediaminetetraacetic acid (EDTA) tube and centrifuged at 3000 × g for 5 min. at 4°C. The supernatant was collected and immediately frozen at −80°C.

### Isolation and purification of exosomes

Exosomes were isolated from tissue homogenate, human and mouse plasma, and cell culture supernatant using ExoCapTM Exosome Isolation and Enrichment kit with composite beads recognizing the exosome surface antigens of CD9, CD63, CD81 and EpCAM (JSR Micro Materials Innovation, Sunnyvale, CA, USA) by following the manufacturer's protocol. Tumour tissues and normal colon tissues were homogenized using Dounce Glass Tissue Grinder Homogenizer in 1 ml lysis buffer (0.1 M Tris.HCI, pH 7.4 and 5 mM EDTA, pH 8.0) containing protease inhibitor cocktail (Roche, Basel, Switzerland). Plasma and cell supernatant were collected by centrifugation at 450 × g for 5 min. The isolated exosomes were purified using sucrose density gradients as previously described 10.

### Transmission electron microscopy of exosomes

Exosomes were directly adsorbed onto carbon/formvar transmission electron microscopy (TEM) grids (400 mesh copper) and dried for 15 min. at room temperature (RT). After rinsing the grids in blocking buffer for 1 hr, the grids were placed into rabbit anti‐human GPC1 antibody (1:200 dilution; Santa Cruz, Dallas, TX, USA) for overnight at 4°C. The grids were incubated with 5% foetal calf serum (FCS) as a negative control. After rinsing with 1× PBS for three times, the grids were incubated with goat‐anti‐rabbit secondary antibody attached with 10‐nM gold particles (Abcam, Cambridge, UK) for 2 hrs at RT. After rising with 1× PBS for three times, the grids were floated in 2.5% glutaraldehyde for 15 min. followed by rinsing in distilled water, drying, and staining with 2.0% uranyl acetate. After further drying, the grids were photographed under JEM‐1100 transmission electron microscope (JEOL, Tokyo, Japan).

### Flow cytometry analysis of exosomes

One milligram of exosomes were incubated with 300 μl of aldehyde/sulphate latex beads (Invitrogen Inc., Carlsbad, CA, USA) for 20 min. at RT with continuous rotation. After dilution with 1× PBS and stopping reaction with 100 mM glycine and 2% bovine serum albumin (BSA) in PBS for 30 min. at RT, exosomes‐bound beads were washed once in 1× PBS containing 2% BSA and centrifuged for 1 min. at 14,800 × g and blocked with 10% BSA for 30 min. The re‐suspended beads were incubated with anti‐GPC1 antibody (Santa Cruz) for 30 min. at 4°C with rotation. After centrifugation, washing and re‐suspension, beads were incubated with Alexa‐488‐tagged secondary antibodies (Life Technologies, Carlsbad, CA, USA) for 30 min. at 4°C with rotation. Secondary antibody incubation alone was used as control and to gate the beads with GPC1‐bound exosomes. The percentage of beads with GPC1^+^ exosomes was calculated.

### Western blot analysis

Tumour tissues and cells were homogenized as described above and the homogenate was used for Western blot analysis 16. The exosome pellets were resuspended in 1× PBS containing protease inhibitor cocktail (Roche). The protein concentration was determined by a modified Bradford Assay (Bio‐Rad Laboratories, Hercules, CA, USA). Tissue homogenate and exosome sample preparations containing 5 μg protein were loaded per well. The membrane was blocked with 5% non‐fat dried milk for 1 hr and incubated overnight at 4°C with anti‐GPC1 antibody, anti‐CD63 antibody and anti‐β‐Actin Antibody (Santa Cruz), followed by incubation with horseradish peroxidase‐conjugated goat anti‐rabbit IgG (Cell Signaling, Danvers, MA, USA) secondary antibody for 2 hrs at RT. A chemiluminescence substrate (ECL Prime; Amersham, Little Chalfont, UK) was used to visualize the immunoreactive proteins. The images were taken using ChemiDoc XRS^+^ system and analysed using Image Lab 4.1 (Bio‐Rad Laboratories).

### Real‐time amplification of microRNA

Total RNA was extracted from plasma or exosomes from CRC tumour tissues, normal colon tissues and plasma using Trizol reagent (Invitrogen Inc.) by following the manufacturer's instructions. Reverse transcription was conducted using One Step PrimeScript^®^ miRNA cDNA Synthesis kit. Real‐time PCR was performed using SYBR Premix Ex Taq II (TaKaRa, Shiga, Japan) and the Applied Biosystems 7500 Fast Real Time PCR System. The miR‐96‐5p was amplified using forward primer: 5′‐TTTGGCACTAGCACATTTTTGCT‐3′, miR‐149 was amplified using forward primer: 5′‐TCTGGCTCCGTGTCTTCACTCCC‐3′, miR‐182‐5p was amplified using forward primer: 5′‐TTTGGCAATGGTAGAACTCACACT‐3′, and U6 was amplified using forward primer: 5′‐GCTTCGGCAGCACATATACTAAAAT‐3′. The reverse primers for miRNA and U6 were provided with the kit. Relative quantification of miRNA expression was analysed using the comparative CT method (^ΔΔ^CT) method. U6 mRNAs were used as internal control for miRNA expression 17.

### Cell culture

HT‐29 and HCT‐116 are human colon carcinoma cell lines, which were obtained from American Type Culture Collection (Manassas, VA, USA) and cultured in DMEM medium (Invitrogen Inc.) containing 10% FCS, 100 μg/ml of streptomycin and 100 units/ml penicillin at 37°C, 5% CO_2_.

### Construction of miR‐96‐5p and miR‐149 expression vector

The miRNA expression vector was constructed according to our previously published protocol 19. Briefly, the minigene sequences that transcribe miR‐96‐5p (5′‐UUUGGCACUAGCACAUUUUUGCU‐3′) and miR‐149 (5′‐UCUGGCUCCGUGUCUUCACUCCC‐3′) were synthesized. Two complementary oligonucleotides corresponding to the sequences of matured miR‐96‐5p and miR‐149 were annealed to yield the miRNA expression minigene and cloned into the pSilencer‐3.0 vector (Ambion Inc., Austin, TX, USA). The miRNA transcription cassette including the H1 promoter, miRNA minigene, and a tract of six thymidines was subclonded into the pShuttle vector of pEasy‐1 adenovirus packaging system. The adenoviruses that express miR‐96‐5p (AdmiR96) and miR‐149 (AdmiR149) were produced, identified and purified as previously described 18. As a negative control, a 22‐nt sequence with no known target in the human genome was used in place of the miRNA minigene to produce the control virus.

### MTT assay

3‐(4,5‐dimethyl‐2‐thiazolyl)‐2,5‐diphenyl‐2‐H‐tetrazolium bromide (MTT) assay for cell proliferation in AdmiR96 or AdmiR149 infected HT‐29 and HCT‐116 cells was carried out using an established protocol 19. Briefly, cells at 80–90% confluency in a 96‐well plate were infected with control virus or miRNA expressing viruses (10 MOI). Forty‐eight hours later, cells were subjected to MTT assay 19. The OD values were normalized to the cells that were infected with control virus.

### Hoechst staining for apoptotic cells

Cellular apoptosis induced by miRNA infection was observed by Hoechst33342 (Calbiochem, San Diego, CA, USA) staining. A published method for Hoechst staining was adopted 20. Briefly, cells at 50–60% confluency were infected with control virus or miRNA expressing virus. Forty eight hours later, cells were fixed in methanol/acetic acid (3:1) for 10 min. at 4°C, followed by staining with Hoechst33342 (5 μg/ml) for 10 min. at RT. Cells that showed clear condensation under a fluorescence microscope were counted as apoptotic cells.

### Tumour growth experiments

Balb/C nude mice (BALB/c, *nu/nu*) weighing 20–22 g were purchased from the animal centre of Shanghai Biological Science Institution and housed in a controlled barrier facility in the Laboratory Animal Research Center in Central South University. The animal protocol was pre‐approved by Central South University and all animals were handled in compliance with the animal care guidelines of the Chinese Council.

Approximately 1 × 10^6^ HT‐29 or HCT‐116 cells were subcutaneously injected into the right hind limbs of nude mice (*n* = 10 for each group). When the tumour grew to 100 mm^3^ in volume, blank control virus, AdmiR96 or AdmiR149 virus was injected locally (1 × 10^7^ pfu). One week later, viruses (1 × 10^8^ pfu) were intratumourally injected again. Tumour size was measured every 2–3 days. Tumour volume was calculated using the formula: V = (L × W2)/2 (L: tumour length, W: tumour width) 19. Animals were killed 22 days after first virus injection, tumour tissues were excised and blood was collected. The plasma was isolated at 1000 × g for 5 min. at 4°C. The tumour and plasma samples were freezed at −80°C.

### Statistical analysis

Data were analysed using SPSS v18.0 (Chicago, IL, USA) and presented as mean ± S.E. Repeated one‐way anova was used for statistical analysis of tumour growth. One‐way anova or two‐tailed Student's *t*‐test was used for statistical analyses of gene expression data. A *P* < 0.05 was considered statistically significant.

## Results

### Isolation and characterization of GPC1+ exosomes

**Figure 1:**
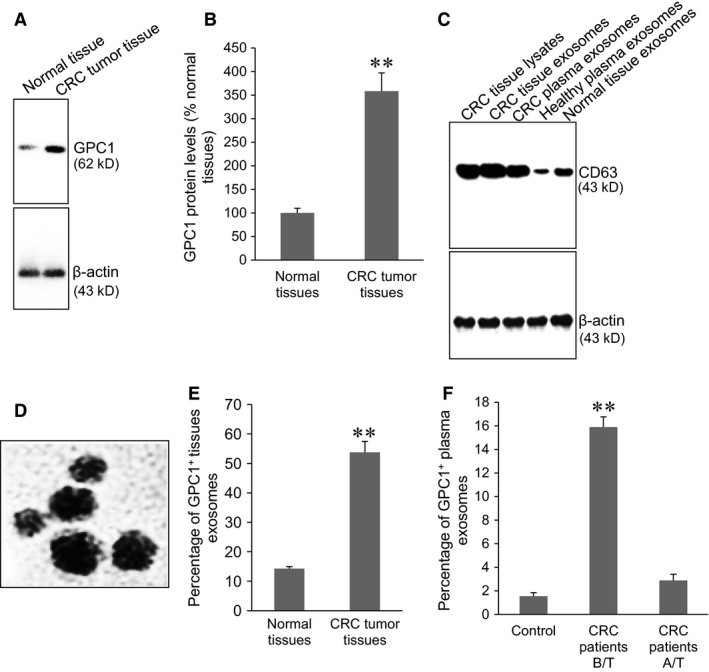
Isolation of GPC1^+^ exosomes. (**A**) Representative Western blots of GPC1 and β‐actin protein expression in tissues. (**B**) Semi‐quantitative analysis of GPC1 protein expression in human normal colon tissues (*n* = 89) and CRC tumour tissues (*n* = 102). **P < 0.001 *versus* normal tissue. (**C**) Representative Western blot of CD63 and β‐actin protein expression in CRC tissue lysates and exosomes. (**D**) A representative TEM of GPC1^+^ exosomes from plasma. (**E**) Cytometry assay of percentage of GPC1^+^ exosomes in CRC tumour tissues (*n* = 102) and normal colon tissues (*n* = 89). **P < 0.001 *versus* normal tissue. (**F**) Cytometry assay of percentage of GPC1^+^ plasma exosomes in CRC patients (*n* = 102) before surgical treatment (B/T), after surgical treatment (A/T), and healthy subjects (control) (*n* = 80). **P < 0.001 *versus* other two groups.

**Figure 2:**
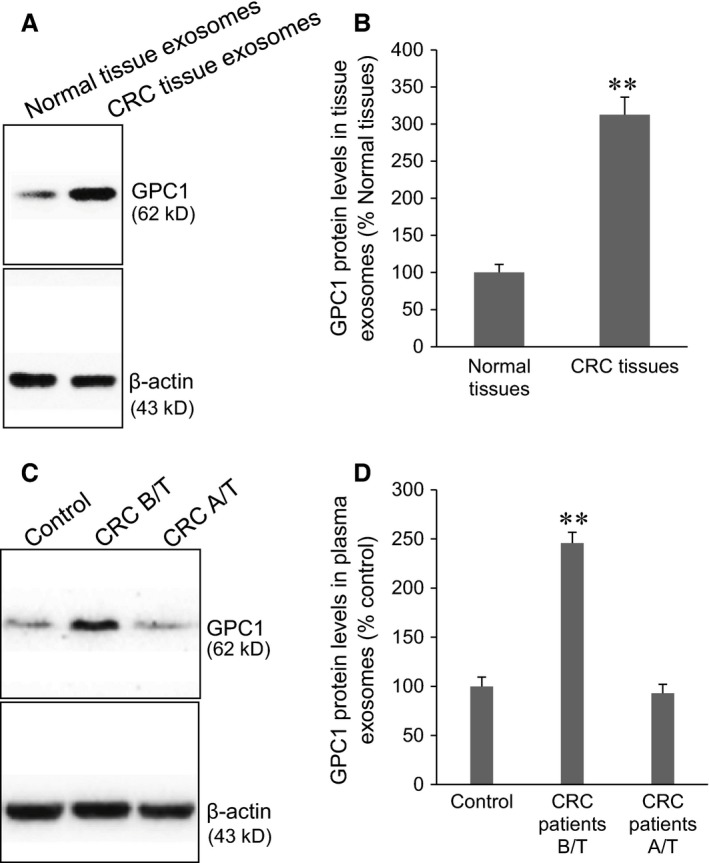
Measure of GPC1 expression in exosomes. (**A**) Representative Western blots of GPC1 protein expression in tumour exosomes and normal tissue exosomes. (**B**) Semi‐quantitative analysis of GPC1 expression in tissue exosomes in (**A**). **P < 0.001 *versus* normal tissue exosomes. *n* = 102 for CRC,* n* = 89 for normal tissue. (**C**) Representative Western blot of GPC1 expression in the plasma of healthy individuals, CRC patients before and after surgical treatment. (**D**) Semi‐quantitative analysis of GPC1 expression in plasma exosomes in (**C**). **P < 0.001 *versus* other two groups. *n* = 102 for CRC,* n* = 80 for healthy control.

A previous study has reported a high expression of GPC1 in human CRC tumour tissues 11. This study confirmed a high GPC1 expression in CRC tumour tissues compared to the normal colon tissues (Fig. 1A, *P* < 0.001). Several exosomal markers, including CD63, Tsg101, Aip1/Alix, β1‐Integrin, CD81, Icam‐1, and Mfg‐E8, have previously been used for rapid confirmation of exosome presence by Western blot analysis, but CD63 is the most abundant protein 21. Same amounts (5 μg) of CRC tumour tissue lysates and exosomes from tumour tissues and plasma were loaded on the same gel. The intensity of the band of CD63 was higher in the exosomes from tumour tissues and plasma of CRC patients than that in the normal tissue lysates and plasma exosomes from healthy individuals (Fig. 1B). TEM revealed that GPC1^+^ exosomes from tumour tissue, plasma of CRC patients and supernatant of HT‐29 and HCT‐116 cells exhibited similar round‐shaped membrane vesicles with diameters of 30–90 nm (Fig. 1C). Several previous studies have reported the biological significance of exosomes in body fluids, including functioning as vehicles for externalization of important intracellular proteins 21, 22, 23. We therefore measured the content of the amount of GPC1^+^ exosomses in tumour tissues and plasma. Cytometry assay showed a significant higher percentage of GPC1^+^ exosomes in CRC tumour tissues than that in the normal colon tissues (Fig. 1D, *P* < 0.001). The fasting blood was collected from healthy individuals as a control and CRC patients before and 2 months after the treatments. The percentage of plasma GPC1^+^ exosomes was significantly higher in CRC patients before surgical treatment than that in healthy controls and in CRC patients after surgical therapy (Fig. 1E). These findings suggest that high levels of plasma GPC1^+^ exosomes is a characteristic of CRC patients, and surgical treatments can lower its percentage. We further validated GPC1 expression in the exosomes (Fig. 2). Western blot showed that GPC1 protein level in tumour exosomes was significantly higher than that in normal colon tissue exosomes (Fig. 2A and B, *P* < 0.001). The GPC1 protein level was significantly higher in the plasma exosomes from CRC patients before surgical treatment than that in the plasma exosomes from healthy individuals and the plasma exosomes after surgical treatment (Fig. 2C and D, *P* < 0.001). These findings suggest that the exosomes from CRC tumour tissues and CRC patients' plasma contain significantly more GPC1 protein than normal colon tissues and the plasma of healthy controls. Also, treatment can downregulate GPC1 protein expression in CRC tumour exosomes.

### miRNA expression in tissues, plasma and exosomes

**Figure 3:**
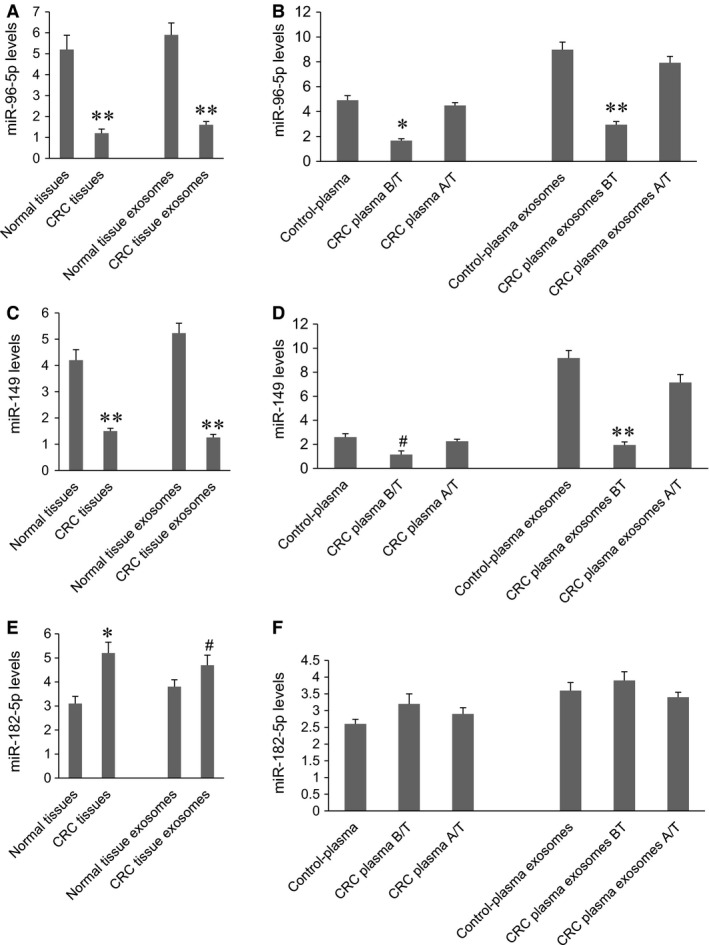
miR‐96‐5p, miR‐149, and miR‐182‐5p expression in tissues, plasma, and exosomes. miRNA expression was measured by real‐time PCR. (**A**) miR‐96‐5p expression in normal colon tissues, CRC tumour tissues, normal tissue exosomes and CRC tumour exosomes. **P < 0.001 *versus* normal tissue or normal tissue exosomes. (**B**) miR‐96‐5p expression in the plasma of healthy individuals, CRC patients before (B/T) and after surgical treatment (A/T), as well as their plasma exosomes. *P < 0.001, **P < 0.001 *versus* other two groups. (**C**) miR‐149 expression in normal colon tissues, CRC tumour tissues, normal tissue exosomes and CRC tumour exosomes. **P < 0.001 *versus* normal tissue or normal tissue exosomes. (**D**) miR‐149 expression in the plasma of healthy individuals, CRC patients before (B/T) and after surgical treatment (A/T), as well as their plasma exosomes. ^#^ *P* < 0.05, **P < 0.001 *versus* other two groups. (**E**) miR‐182‐5p expression in normal colon tissues, CRC tumour tissues, normal tissue exosomes, and CRC tumour exosomes. ^#^ *P* < 0.05, *P < 0.01 *versus* normal tissue or normal tissue exosomes. (**F**) miR‐182‐5p expression in the plasma of healthy individuals, CRC patients before (B/T) and after surgical treatment (A/T), as well as their plasma exosomes. *n* = 102 for CRC,* n* = 89 for normal tissue, *n* = 80 for control.

The expression of miR‐96‐5p, miR‐149 and miR‐182‐5p in CRC tumour tissues, normal colon tissues, plasma and their exosomes was measured by real‐time PCR (Fig. 3). miR‐96‐5p expression was significantly decreased in CRC tumour tissues and CRC tumour exosomes compared to that in normal colon tissues and normal tissue exosomes, respectively (Fig. 3A). The miR‐96‐5p expression was significantly decreased in CRC plasma before surgical treatment compared to that in the plasma of healthy individuals, but the plasma miR‐96‐5p level in CRC patients was significantly normalized after surgery (Fig. 3B, *P* < 0.01). Similarly, miR‐96‐5p expression was significantly decreased in CRC plasma exosomes before surgical treatment compared to that in the plasma exosomes from healthy individuals, but the miR‐96‐5p level in CRC plasma exosomes was normalized after surgery (Fig. 3B, *P* < 0.001). The changes in miR‐149 expression were similar to miR‐96‐5p in tumour tissues and tumour tissue exosomes (Fig. 3C, *P* < 0.001), as well as in CRC plasma and CRC plasma exosome before (*P* < 0.05) and after surgery (*P* < 0.001) (Fig. 3D). miR‐182‐5p expression was significantly increased in CRC tumour tissues (*P* < 0.01) and CRC tumour exosomes (*P* < 0.05) compared to that in normal colon tissues and normal tissue exosomes, respectively (Fig. 3E). However, no significant differences in miR‐182‐5p expression was observed in CRC plasma and plasma exosomes before and after surgical treatment and the plasma of healthy individuals (Fig. 3F).

### Overexpression of miR‐96‐5p and miR‐149 inhibited GPC1 expression, GPC1^+^ exosomes secretion, increased cell apoptosis and inhibited cell proliferation in HT‐29 and HCT‐116 cells

**Figure 4:**
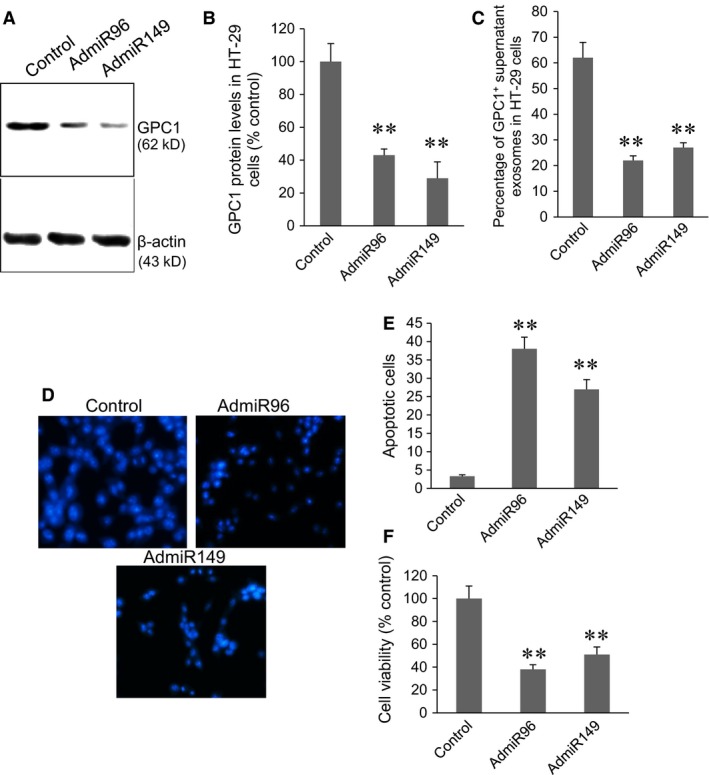
Silencing of miR‐96‐5p and miR‐149 expression in HT‐29 cells. (**A**) Representative Western blot of GPC1 protein expression in control virus (control), AdmiR96, and AdmiR149 infected HT‐29 cells. (**B**) Semi‐quantitative analysis of GPC1 expression in HT‐29 cells in (**A**). (**C**) Cytometry assay of the percentage of GPC1^+^ exosomes in the supernatant of cultured HT‐29 cells infected with viruses. (**D**) Representative Hoechst33342 staining of HT‐29 cells infected with viruses. (**E**) Percentage of apoptotic HT‐29 cells infected with virus. (**F**) MTT assay of cell viability in HT‐29 cells infected with viruses. **P < 0.001 *versus* control, *n* = 4.

HT‐29 and HCT‐116 cells were infected with 10 MOI of control viruses, AdmiR96 or AdmiR149 virus for 48 hrs. In HT‐29 cells, both AdmiR96 and AdmiR149 virus infection significantly inhibited GPC1 protein expression (Fig. 4A and B, *P* < 0.001). Cytometry assay showed that both AdmiR96 and AdmiR149 virus infection significantly decreased the percentage of GPC1^+^ exosomes in the supernatant of cultured HT‐29 cells (Fig. 4C, *P* < 0.001). Cell apoptosis was visualized by Hoechst33342 staining (Fig. 4D). Both AdmiR96 and AdmiR149 virus infection significantly induced cell apoptosis in HT‐29 cells compared to control virus infection (Fig. 4E, *P* < 0.001). MTT assay showed that both AdmiR96 and AdmiR149 virus infection significantly decreased the cell proliferation in HT‐29 cells (Fig. 4F).

**Figure 5:**
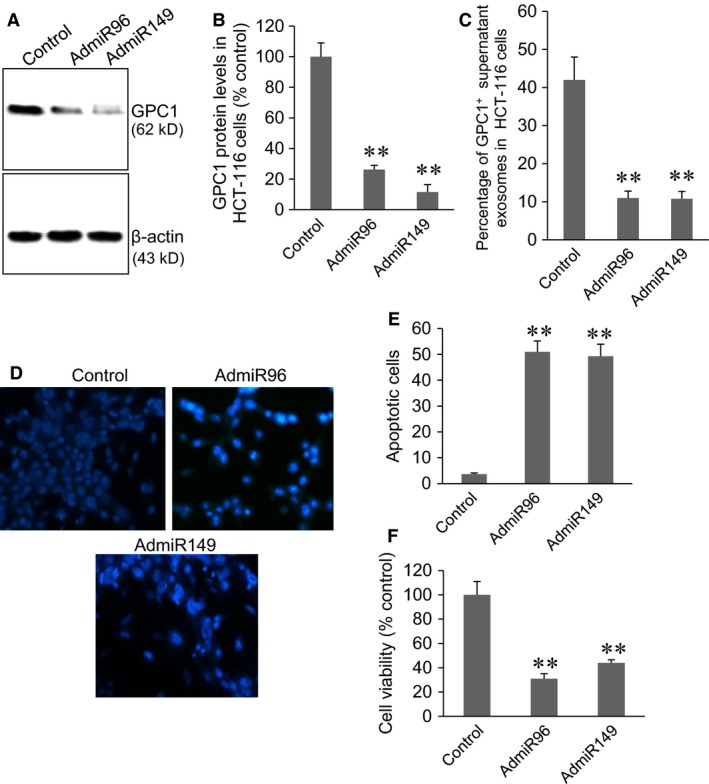
Silencing of miR‐96‐5p and miR‐149 expression in HCT‐116 cells. (**A**) Representative Western blot of GPC1 protein expression in control virus (control), AdmiR96, and AdmiR149 infected HCT‐116 cells. (**B**) Semi‐quantitative analysis of GPC1 expression in HCT‐116 cells in (**A**). (**C**) Cytometry assay of percentage of GPC1^+^ exosomes in the supernatant of viruses‐infected HCT‐116 cells. (**D**) Representative Hoechst33342 staining of HCT‐116 cells infected with viruses. (**E**) Percentage of apoptotic HCT‐116 cells infected with virus. (**F**) MTT assay of cell viability in HCT‐116 cells infected with viruses. **P < 0.001 *versus* control, *n* = 4.

In HCT‐116 cells, both AdmiR96 and AdmiR149 virus infection significantly inhibited GPC1 protein expression (Fig. 5A and B, *P* < 0.001), significantly decreased the percentage of GPC1^+^ exosomes in the supernatant of cultured HCT‐116 cells (Fig. 5C, *P* < 0.l001), significantly induced cell apoptosis (Fig. 5D and E, *P* < 0.001), and significantly decreased the cell proliferation (Fig. 5F) compared to control virus infection.

### Administration of AdmiR96 and AdmiR149 viruses inhibited GPC1 expression, GPC1^+^ tumour tissue exosomes and tumour growth in HT‐29 and HCT‐116 xenograft tumour models

**Figure 6:**
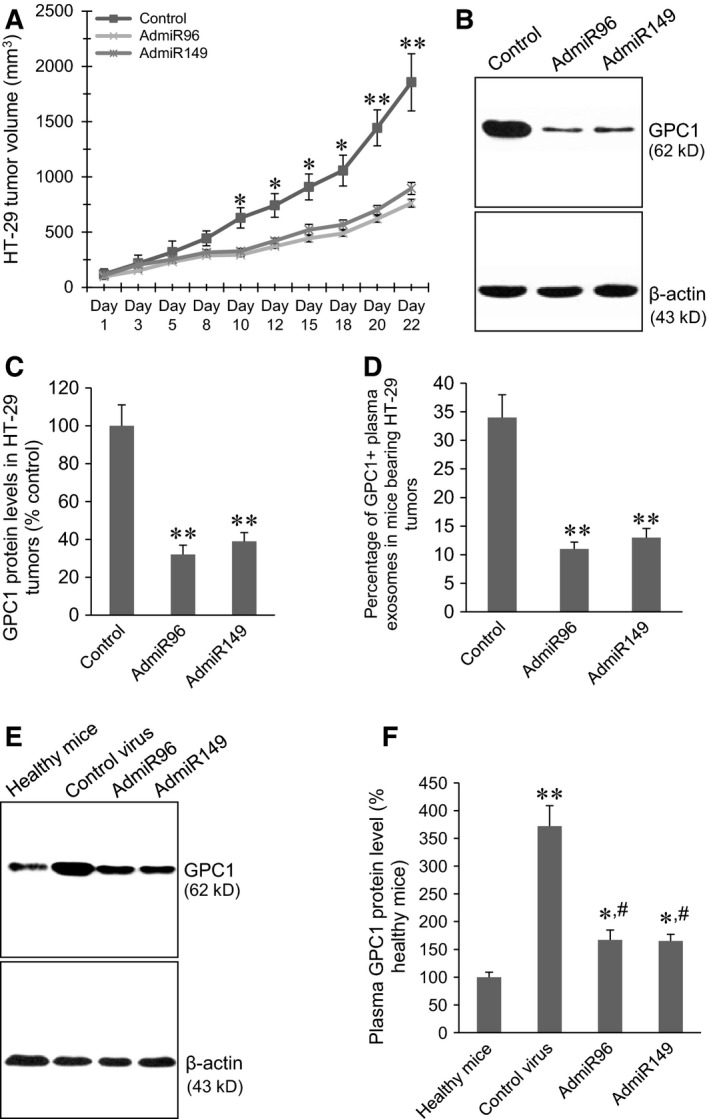
Inhibition of GPC1 expression and secretion of GPC1^+^ exosomes suppressed xenograft HT‐29 tumour growth. (**A**) Injection of AdmiR96 and AdmiR149 viruses inhibited xenograft HT‐29 tumour growth. The control mice were injected with control virus. (**B**) Representative Western blots of GPC1 protein expression in the HT‐29 tumour tissues. (**C**) Semi‐quantitative analysis of GPC1 expression in HT‐29 tumours measured by Western blot. **P < 0.001 *versus* control, *n* = 10. (**D**) Cytometry assay of the percentage of GPC1^+^ exosomes in the plasma of mice bearing HT‐29 tumours. **P < 0.001 *versus* control, *n* = 10. (**E**) Representative Western blots of plasma GPC1 protein levels in mice bearing HT‐29 xenograft tumours and healthy mice. (**F**) Semi‐quantitative analysis of plasma GPC1 expression in mice bearing HT‐29 xenograft tumours and healthy mice. **P < 0.001 *versus* healthy mice, *P < 0.05 *versus* healthy mice, ^#^ *P* < 00.01 *versus* mice bearing HT‐29 xenograft tumours injected with control virus, *n* = 10.

Xenograft HT‐29 and HCT‐116 tumour models were established in nude mice by subcutaneous injection of HT‐29 and HCT‐116 cells into the right hind limbs. The control, AdmiR96 and AdmiR149 viruses were intratumourally injected twice at two different dates. In the HT‐29 xenograft tumour models, both AdmiR96 and AdmiR149 virus injection significantly inhibited tumour growth beginning at day‐10 post first injection (Fig. 6A, *P* < 0.01, *P* < 0.001). Western blot showed that both AdmiR‐96 and AdmiR149 virus injection significantly inhibited GPC1 protein expression in the HT‐29 tumour tissues (Fig. 6B and C). Cytometry assay showed that both AdmiR96 and AdmiR149 virus injection significantly decreased the percentage of GPC1^+^ exosomes in the plasma of mice bearing HT‐29 tumours (Fig. 6D, *P* < 0.001). Western blot showed that plasma GPC1 protein levels in mice bearing HT‐29 tumours were significantly increased compared to the healthy mice (*P* < 0.001). Both AdmiR‐96 and AdmiR149 virus injection significantly decreased the plasma GPC1 protein levels in mice bearing HT‐29 tumours (Fig. 6E and F).

**Figure 7:**
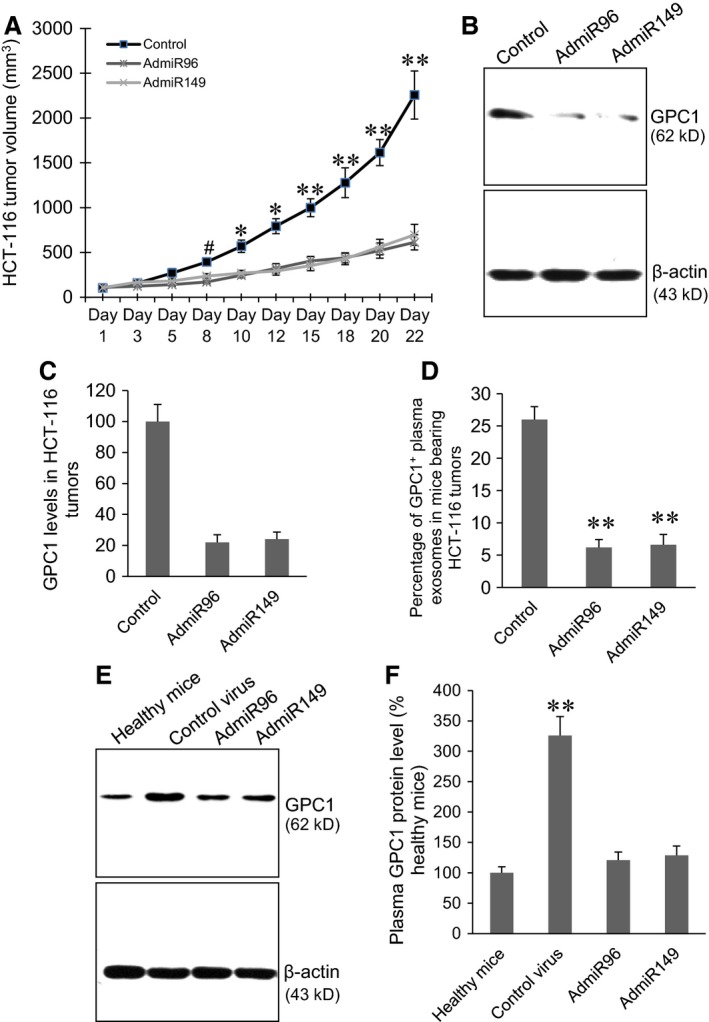
Inhibition of GPC1 expression and secretion of GPC1^+^ exosomes suppressed xenograft HCT‐116 tumour growth. (**A**) Injection of AdmiR96 and AdmiR149 viruses inhibited xenograft HCT‐116 tumour growth. The control mice were injected with control virus. #*P* < 0.05, *P < 0.01, **P < 0.001 *versus* AdmiR96 and AdmiR149 group, *n* = 10. (**B**) Representative Western blot of GPC1 protein expression in the HCT‐116 tumour tissues. (**C**) Semi‐quantitative analysis of GPC1 expression in HCT‐116 tumours measured by Western blot. **P < 0.001 *versus* control, *n* = 10. (**D**) Cytometry assay of percentage of GPC1^+^ exosomes in plasma of mice bearing HCT‐116 tumours. **P < 0.001 *versus* control, *n* = 10. (**E**) Representative Western blots of plasma GPC1 protein levels in mice bearing HCT‐116 tumours and healthy mice. (**F**) Semi‐quantitative analysis of plasma GPC1 expression in mice bearing HCT‐116 xenograft and healthy mice. **P < 0.001 *versus* all other groups, *n* = 10.

In the HCT‐116 xenograft tumour models, both AdmiR96 and AdmiR149 virus injection significantly inhibited tumour growth beginning at day‐8 post first injection (Fig. 7A, *P* < 0.05, *P* < 0.01, *P* < 0.001), significantly inhibited GPC1 protein expression in the HCT‐116 tumour tissues (Fig. 7B and C), and significantly decreased the percentage of GPC1^+^ exosomes in the plasma of mice bearing HCT‐116 tumours (Fig. 7D, *P* < 0.001). Western blot showed that plasma GPC1 protein levels in mice bearing HCT‐116 tumours were significantly increased compared to the healthy mice (*P* < 0.001). Both AdmiR‐96 and AdmiR149 virus injection significantly decreased the plasma GPC1 protein levels in mice bearing HCT‐116 tumours (Fig. 7E and F).

## Discussion

Exosomes are secreted from various cells including tumour cells. Therefore, identifying cancer‐specific exosomes is important for the diagnosis and prognosis of cancer patients; however, isolation of cancer‐specific exosomes is difficult due to a lack of specific markers. A recent study suggested that GPC1 is a specific marker of exosomes in pancreatic cancer 10. Glypican‐1 has been observed to be overexpressed in the CRC tumour tissues 11. However, whether GPC1 is also a specific marker for the exosomes of CRC tumours has not been addressed. In the present study, we identified exosomes in the plasma and tumour tissues, which exhibited enriched GPC1 protein level in CRC patients for the first time. We further provided evidence for the secretion of GPC1 positive exosomes from CRC tumour cells *in vitro* and *in vivo*. Moreover, we identified miR‐96‐5p and miR‐149 as the regulatory factors of GPC1 expression in the tumour tissues and tumour specific exosomes and their roles in the cell proliferation, apoptosis and tumour growth.

Consistent with a previous report 11, this study observed GPC1 overexpression in CRC tumour tissues. Exosomes were then isolated from tumour tissue lysates, normal colon tissue lysates, the plasma from healthy individuals, and CRC patients before and after surgical treatment using a commercial available kit. The exosomes were confirmed by high expression of exosome specific marker CD63 and TEM imaging, suggesting a successful isolation of exosomes. Furthermore, GPC1 positive exosomes were purified using cytometry. The percentage of GPC1^+^ exosomes in tumour tissues is about fourfold higher than that in the normal tissues. The percentage of GPC1^+^ exosomes in the plasma of CRC patients was over 10‐fold higher than that in the plasma of healthy subjects. Importantly, the GPC1 protein levels in GPC1 positive exosomes from the tumour tissues and plasma of CRC patients was significantly higher than that from peritumoural tissues and plasma of healthy subjects, respectively. Moreover, the secretion of GPC1^+^ exosomes was validated in the plasma of mice bearing HT‐29 and HCT‐116 tumours. These findings suggest that GPC1 positive exosomes is a CRC specific biomarker. In addition, both the GPC1 positive exosomes and GPC1 protein expression in GPC1 positive exosomes were normalized 2 months after surgery. This observation suggests that GPC1 positive exosomes is also an indicator of the therapeutic efficacy in CRC patients.

Although a previous study reported the successful isolation of GPC1 positive exosomes from pancreatic cancer and suggested it as a specific marker for the diagnosis and prognosis of pancreatic cancer 10, the regulations of GPC1 expression in both the tumour cells and the exosomes were not addressed. The roles of miRNA in post transcriptional regulation of mRNA have been extensively studied. In pancreatic cancer, miR‐96‐5p suppressed GPC1 expression 12, and miRNA‐149 is also thought to regulate GPC1 expression 13. In this study, we observed a loss of miR‐96‐5p and miR‐149 expression in the tumour tissues and plasma of CRC patients and GPC1 positive exosomes from CRC tumour tissues and plasma of CRC patients before surgical treatment. Importantly, the plasma miR‐96‐5p and miR‐149 levels were restored within 2 months after surgery. The changes in miR‐96‐5p and miR‐149 correlated with the changes in GPC1 protein expression and GPC1^+^ exosomes from the tumour tissues and plasma of CRC patients. Although the expression of miR‐182‐5p was elevated in the tumour tissues and GPC1 positive exosomes from CRC patients, its expression in the plasma and plasma GPC1^+^ exosomes was not significantly changed. We further investigated the relationship between miR‐96‐5p and miR‐149 expression and GPC1 expression in two CRC cell lines. Overexpression of miR‐96‐5p and miR‐149 significantly inhibited GPC1 expression in both HT‐29 and HCT‐116 cells. Also, overexpression of miR‐96‐5p and miR‐149 significantly decreased the secretion of GPC1 positive exosomes from HT‐29 and HCT‐116 cells *in vitro* as well as xenograft HT‐29 and HCT‐116 tumours *in vivo*. The growth of xenograft HT‐29 and HCT‐116 tumours increased plasma GPC1 protein levels while overexpression of miR‐96‐5p and miR‐149 in xenograft tumours significantly decreased plasma GPC1 protein levels in mice. These findings suggest that miR‐96‐5p and miR‐149 not only regulate GPC1 expression in tumour cells, but also regulate the secretion of GPC1 positive exosomes and the enrichment of GPC1 in the exosomes.

Previous studies have observed the roles of GPC1 in tumour growth and angiogenesis 5, 6, 7 through promotion of FGF‐FGFR activation and signalling 9. This study demonstrated that overexpression of miR‐96‐5p and miR‐149 downregulated GPC1 expression in HT‐29 and HCT‐116 cells and xenograft HT‐29 and HCT‐116 tumours as well as decreased the secretion of GPC1 positive exosomes and subsequently induced cell apoptosis and inhibited cell proliferation *in vitro* and tumour growth *in vivo*. Although miR‐96‐5p and miR‐149 may regulate many genes and these genes may also be involved in the tumour growth, the regulatory effect of these two miRNAs on the secretion of GPC1 positive exosomes supports the feasibility of using CRC cell cultures for future mechanism studies of exosome release from cells and obtaining large amounts of exosomes for therapeutic purposes. Also, GPC1 positive exosomes might have promising roles as diagnostic and therapeutic targets.

In conclusion, CRC is the second leading cause of cancer‐related deaths worldwide. However, there are no clinically available biomarkers for effective targeting therapies and early diagnosis of CRC. In addition, the molecular mechanisms for the progression of CRC remains to be elucidated. In this study, we provided initial evidence that significantly elevated GPC1^+^ exosomes are present in the plasma of CRC patients and can be secreted from CRC tumour cells. Together with GPC1 expression and GPC1^+^ exosomes, miR‐96‐5p and miR‐149 could be markers for the diagnosis, evaluation of therapeutic efficacy, and targets for molecular therapy of CRC.

## Conflict of interest

The authors declared no conflict of interest.
